# Advances in multi-omics research on neuroblastoma

**DOI:** 10.3389/fped.2026.1739376

**Published:** 2026-04-16

**Authors:** Yubing Wang, Zhifei Zhao, Jinbin Wang, Shujie Song, Chengmin Zhao, Chao Qv, Hongting Lu

**Affiliations:** 1 Department of Pediatric Surgical Oncology, Affiliated Women and Children’s Hospital of Qingdao University, Qingdao, Shandong, China; 2 Department of Thoracic and Cardiovascular Surgery, Weifang Second People’s Hospital, Weifang, Shandong, China; 3 Department of Hepatobiliary and Pancreas, Affiliated Hospital of Qingdao University, Qingdao, Shandong, China

**Keywords:** multi-omics integration, neuroblastoma, pathomics, radiomics, transcriptomics

## Abstract

Neuroblastoma is the most common extracranial solid tumor in children, presenting significant challenges in diagnosis and treatment due to its highly heterogeneous clinical manifestations and complex genetic background. In recent years, advances in transcriptomics have played a pivotal role in this field, not only aiding in the identification of molecular subtypes of tumors but also revealing potential mechanisms of drug resistance. Through comprehensive gene expression profiling and single-cell sequencing technology, researchers have deeply analyzed key interaction nodes within the metabolic-immune microenvironment, providing a theoretical basis for developing targeted therapeutic strategies. Concurrently, radiomics, leveraging imaging techniques such as MRI, PET-CT, and CT, quantitatively assesses the morphological and metabolic characteristics of tumors. This enables non-invasive prediction of MYCN amplification status, evaluation of bone marrow metastasis risk, and prognostic stratification, thereby supporting dynamic disease monitoring. In pathology, artificial intelligence technology is widely applied in the analysis of digital pathology images. It effectively identifies cellular diversity and immune microenvironment features in tissues, enhancing diagnostic accuracy and assisting in predicting potential gene mutations. More importantly, integrating transcriptomics, radiology, and pathology data through multi-omics approaches overcomes the limitations of single data types. This integration constructs more precise disease classification models and facilitates the development of personalized treatment plans. This review emphasizes the critical roles of transcriptomics, radiomics, digital pathology analysis, and multi-omics fusion strategies in enhancing diagnostic precision for neuroblastoma and optimizing treatment decisions.

## 1 Introduction

Neuroblastoma (NB) is the most common extracranial solid tumor in children, accounting for approximately 8%–10% of all childhood malignancies, with its peak incidence occurring predominantly during infancy and early childhood (1–3). This malignancy originates from precursor cells of the sympathetic nervous system and exhibits pronounced heterogeneity, ranging from low-grade malignant spontaneously regressing forms to highly aggressive and recurrent high-risk subtypes. Although recent therapeutic advances have improved survival rates for some patients, the 5-year survival rate remains below 50% for high-risk patients (4). The existing classification system, based on the International Neuroblastoma Staging System (INSS) and traditional molecular markers, falls short of comprehensively capturing the tumor's biological characteristics. Consequently, the development of precise subtyping strategies that integrate multi-omics data—including transcriptomics, radiomics, and pathology—is increasingly emerging as a key research direction for improving the prognosis of high-risk patients and achieving personalized treatment.

Recent years have witnessed rapid advances in multi-omics technologies, propelling research in Neuroblastoma. Transcriptomics (5, 6), utilizing bulk RNA-seq and single-cell RNA-seq (scRNA-seq), not only delineates gene expression profiles within NB but also uncovers distinct expression patterns across molecular subtypes and their associated aberrant signaling pathways. Concurrently, it has also illuminated the regulatory roles of long non-coding RNAs (lncRNAs) and other non-coding RNAs in tumor development and progression. Radiomics (7–9) utilizes multi-modal imaging data from MRI, PET-CT, and other techniques. Through advanced image processing and deep learning algorithms, it extracts a series of quantifiable imaging features. These features reflect the tumor's morphological structure and metabolic activity, are closely correlated with molecular subtypes and patient prognosis, and can provide clinicians with non-invasive, real-time dynamic monitoring. In the realm of Pathomics (10), the deep integration of digital pathology with artificial intelligence has enabled the high-throughput quantitative analysis of whole-slide scan images. This allows for more precise interpretation of traditional pathological features such as mitotic index and cellular polymorphism. Simultaneously, this technology provides methodological support for revealing the spatial heterogeneity of the tumor microenvironment.

Multi-omics technologies comprehensively decode the intrinsic relationships among molecular features, imaging manifestations, and pathological architectures of neuroblastoma by integrating genomic, radiological, and histopathological data, thereby transcending the limitations of unimodal data analysis. This interdisciplinary paradigm not only facilitates more precise tumor subtyping and disease progression prediction but also reveals novel therapeutic targets, thereby advancing personalized precision medicine. This review aims to synthesize recent advances in Transcriptomics, Radiomics, and Pathomics for neuroblastoma research, critically examining their respective strengths, current applications, and prevailing challenges. Furthermore, it explores the transformative potential of multi-omics data integration in clinical translation, ultimately providing novel theoretical frameworks and practical guidance to improve clinical outcomes in pediatric patients.

## 2 Transcriptomics

Transcriptomics serves as a critical approach for investigating transcriptional-level changes in genes under specific biological conditions, encompassing established techniques including conventional microarray analysis, RNA sequencing, and the rapidly advancing single-cell RNA sequencing; a comprehensive summary of these methodologies is presented in Table 1. In neuroblastoma research, the extensive application of transcriptomic technologies has profoundly deepened the understanding of its molecular characteristics. These advances have played an instrumental role in refining molecular classification, prognostic assessment, and the exploration of targeted therapeutic strategies.

**Table 1:** Summary of transcriptomics research advances.

Author & Year	Research Objective	Design	Sample Size	Key Findings
Sen et al. (11)	Identify recurrent *cis*-regulatory alterations in high-risk neuroblastoma	Bioinformatics analysis	96	Identified 1,043 genes, with *TFAP2B* and *PTPRH* validated as tumor suppressor candidates
Hu et al. (18)	Classify MYCN non-amplified neuroblastoma subtypes and build a prognostic model	Bioinformatics analysis	1,566	Stratified into three subtypes (poor prognosis, MYCN-like, immune-infiltrated) and developed a 21-gene prognostic model
Gupta et al. (19)	Develop a prognostic gene signature for neuroblastoma	Bioinformatics analysis	243	21-gene risk score independently predicted survival (AUC >0.81) and improved accuracy when combined with clinical variables
Luo et al. (20)	Evaluate pyroptosis-related gene networks for immunotherapy response prediction	Bioinformatics analysis	946	Higher pyroptosis scores correlated with better prognosis and immunotherapy response; etoposide enhanced pyroptosis sensitivity
Ke et al. (21)	Identify prognostic biomarkers via WGCNA	Bioinformatics analysis	721	Constructed a model based on *DHFR*, *GMPS*, and *E2F3*; high-risk groups showed oxaliplatin sensitivity
Ognibene et al. (27)	Assess *H2AFX* as a prognostic biomarker and its oncogenic mechanism	Bioinformatics analysis	834	*H2AFX* overexpression linked to poor prognosis; *in vitro* experiments confirmed its pro-tumor role
Hussein et al. (28)	Identify biomarkers and therapeutic targets via multi-omics integration	Bioinformatics analysis	99	Identified 3 transcription factors and 7 miRNAs as candidate biomarkers, some linked to tumorigenesis
Yan et al. (29)	Build a malignant cell marker-based prognostic model for high-risk neuroblastoma	Bioinformatics analysis	366	Developed a 6-gene model (e.g., *MAPT*, *MEG3*) to distinguish ultra-high-risk from standard high-risk patients
Hong et al. (30)	Explore prognostic and mechanistic roles of ICD-related genes in neuroblastoma	Bioinformatics analysis	1,260	*ELAVL3* identified as a key regulator; its silencing suppressed MYCN and enhanced NK cell antitumor activity
Olsen et al. (37)	Investigate SCP-like malignant precursor cells in neuroblastoma heterogeneity	Bioinformatics analysis	24	Identified chromosome 17-aberrant SCP-like subclones, suggesting neural crest-derived tumor evolution
Yang et al. (48)	Construct ganglioside-related prognostic models and explore TME interactions	Bioinformatics analysis	871	B3GALT4 inhibited metastasis; risk model predicted immune evasion and chemotherapy sensitivity
Wang et al. (51)	Prognostic value of neurodevelopmental cell communication pathways in neuroblastoma	Bioinformatics analysis	22	Identified the BMP7-(BMPR1B-ACVR2B) axis as a promoter of tumor migration, with high BMP7 expression in metastatic samples
Li et al. (52)	Pan-cancer analysis of senescence-related CAFs and their predictive value in NB	Bioinformatics analysis	1,617	Built an SCRS prognostic model; low-risk groups had enriched immune infiltration, with JAK1 as a key gene linked to tumor malignancy
Kojima et al. (54)	Single-cell sequencing of CTC molecular features in neuroblastoma	Bioinformatics analysis	14	CTCs harbored ALK mutations/MYCN amplification; inter-subgroup heterogeneity suggested diverse metastatic mechanisms
Grossmann et al. (55)	Characterize chemotherapy-resistant persister cells in high-risk neuroblastoma	Bioinformatics analysis	40	Resistant cells showed suppressed MYCN activity, NF-*κ*B activation, and TME interactions promoting survival

### 2.1 Molecular subtyping

Research has revealed that neuroblastoma exhibits significant molecular heterogeneity, primarily driven by somatic copy number alterations (SCNAs). Sen et al. (11) discovered that SCNAs synergistically interact with cis-regulatory mutations to influence gene dosage effects, and identified candidate tumor suppressor genes such as TFAP2B and PTPRH. The Körber team (12) further demonstrated that all neuroblastoma subtypes originate from mitotic abnormalities during the first trimester, with high-risk tumors often exhibiting telomere maintenance mechanisms and a prolonged evolutionary course. Tumor duration has been confirmed as an independent prognostic factor. Recent studies also indicate that mitotic dysregulation is a core feature of MYCN-driven tumor initiation, with preneuronal ganglia already showing overexpression of mitotic genes similar to that observed in established tumors (13).

### 2.2 Prognostic modeling and treatment response prediction

Chromosomal instability (CIN) is a critical molecular feature of high-risk neuroblastoma subtypes. Ognibene et al. (14) proposed a CIN quantification metric based on the number of chromosomal breakpoints, termed the chromosome breakage index. This index shows a significant negative correlation with patient survival (HR = 2.34, *p* < 0.001), and particularly in MYCN non-amplified low-risk patients, it effectively identifies subgroups with a high risk of recurrence (AUC = 0.82). Studies on genetic heterogeneity reveal that high-risk groups are enriched for mutations in the RAS pathway (CSF1R/MSH6) and NF1 deletions (which can activate the MAPK pathway), while low-risk groups more frequently exhibit abnormalities in epigenetic regulatory genes (EP300/TET2) (15–17). Hu et al. (18) developed a 420-gene classifier that stratified MYCN non-amplified tumors into AURKA-driven, inflammatory, and intermediate subtypes (with 93% accuracy). The 21-gene prognostic signature developed by Gupta et al. (19) achieved an AUC > 0.81 in the validation cohort. In immune microenvironment quantification, Luo et al. (20) proposed a pyroptosis-related score that improved immunotherapy response rates by 2.1-fold. Furthermore, through WGCNA analysis, researchers found significant activation of cell cycle pathways in the high-risk group, accompanied by an immunosuppressive state (21). Additionally, the 5-gene PCR assay developed by Asgharzadeh et al. (22) enables monitoring of minimal residual disease, effectively predicting recurrence risk even in patients who achieved complete remission (HR = 1.89, *p* = 0.01).

### 2.3 Multi-omics mechanistic insights

The progression of neuroblastoma is dually regulated by epigenetic abnormalities and the metabolic-immune microenvironment. Studies demonstrate that multi-exon deletions in the ATRX gene lead to genomic instability via the alternative lengthening of telomeres (ALT) mechanism and are significantly associated with 11q chromosome deletion (23, 24). Additionally, METTL3 maintains telomere stability through m6A modification of the TERRA long non-coding RNA, and its specific inhibitor has been shown to enhance tumor sensitivity to chemotherapy (25). At the epigenetic regulatory level, EZH1 forms a complex with the MYCN protein to co-regulate the expression of key cell cycle genes such as TYMS and CCNA1, while overexpression of H2AFX not only indicates poor prognosis but may also serve as a therapeutic target against MYCN (26, 27). Multi-omics integration further reveals disease regulatory networks. Hussein's team constructed a MYCN-driven transcriptional and post-transcriptional regulatory network, identifying 3 key transcription factors and 7 miRNA markers (28). Yan et al. (29) developed an MMGS model based on single-cell sequencing, enabling precise identification of ultra-high-risk patient subgroups characterized by metabolic dysregulation and immune suppression. Hong et al. (30) found that reduced immune infiltration in high-risk patients is closely linked to the ELAVL3-MYCN regulator*y* axis. Metabolic studies indicate that MYCN-amplified tumors frequently exhibit abnormalities in glycerolipid and purine metabolic pathways (31), while EF2K inhibitors significantly inhibit tumor invasion by blocking the cell cycle (32). Notably, the therapeutic potential of viral therapies in neuroblastoma has recently emerged. Research shows that the Zika virus activates the IRE1-UPR pathway via its NS4B protein, triggering TNF signaling activation and lipid metabolism reprogramming to exert oncolytic effects (33). Meanwhile, enterovirus EV-A71 induces calcium signaling dysregulation and tumor cell apoptosis through the NF-κB pathway (34). Collectively, these findings systematically elucidate the multi-layered driving mechanisms of neuroblastoma, spanning genetic mutations, epigenetic regulation, and metabolic-immune interactions. This provides both a theoretical foundation and potential clinical translation pathways for integrated epigenetic interventions, metabolic modulation, and viral therapies.

### 2.4 Translational applications of targeted therapies

Precision treatment strategies based on molecular subtyping have markedly enhanced the clinical efficacy of neuroblastoma therapy; for ALK-low expressing tumors, combining ALK inhibitors with CAR-T therapy increased response rates by 47% (35), while the third-generation ALK inhibitor lorlatinib combined with chemotherapy achieved complete tumor regression in preclinical models (36). In metabolic interventions, tumor subgroups with high DHODH expression are associated with poor prognosis (5-year survival <10%), indicating that targeting DHODH combined with temozolomide may be effective for this subtype (37); additionally, inhibiting the LIN28B-mediated glutathione metabolic pathway shows potential to reverse chemoresistance and offers novel approaches to overcome drug tolerance (38). For epigenetic interventions, the METTL3 inhibitor STM2457 effectively suppressed tumor proliferation and induced maturation differentiation by regulating m6A modificatio (39), while HDAC inhibitors upregulated MHC class I expression to enhance tumor immunogenicity, providing theoretical support for immunotherapy combinations (40). Collectively, the continuous refinement of these targeted strategies expands neuroblastoma treatment options and establishes a solid foundation for individualized precision therapy.

### 2.5 Cell subpopulations and evolutionary trajectories

Research has identified a subset of SCP-like cells harboring chromosome 17q gain in neuroblastoma. These cells exhibit simultaneous activation of proliferative and apoptotic signaling pathways, suggesting their potential role as key initiating clones in tumorigenesis and malignant transformation (41). Further studies reveal that tumor cells dynamically interconvert between undifferentiated mesenchymal and adrenergic-differentiated states, a process governed by a super-enhancer-driven PHOX2B/HAND2 transcriptional regulatory network. Genes including ASCL1 and SOX11 have been identified as critical molecular switches regulating this phenotypic plasticity (42) These research advances not only illuminate the evolutionary trajectories of distinct cellular subpopulations within the neuroblastoma microenvironment but also provide fundamental theoretical frameworks and methodological tools for unraveling tumor drug resistance mechanisms and developing targeted interventions based on cellular fate modulation.

### 2.6 Tumor microenvironment (TME) profiling

The neuroblastoma tumor microenvironment drives disease progression through multifaceted immunosuppressive mechanisms, as single-cell data reveal highly proliferative tumor subclusters secrete immunosuppressive factors like IL-10 to recruit regulatory T cells and enhance local immune evasion—notably, the Treg marker MSRS serves as both an independent prognostic indicator and guides clinical application of PLK1 inhibitors (43). Microenvironmental features vary markedly across molecular subtypes: mesenchymal-like subtypes exhibit immune-activation potential with abundant naïve T cells, whereas MYCN-amplified tumors display immunosuppressive traits characterized by T-cell dysfunction and elevated immune checkpoint expression (44). Pervasive T-cell exhaustion involves abnormal TOX gene activation via promoter hypomethylation, upregulating co-inhibitory receptors (PD-1/Tim-3), while metabolic disturbances like oxidative stress further impair T-cell functionality (45, 46). Though CAR-T therapy shows efficacy against GD2-positive tumors, tumor cells develop escape mechanisms through GD2 downregulation, Treg recruitment, and aberrant ganglioside metabolism (47, 48). Promising therapeutic strategies include dual TIGIT/PD-L1 blockade inducing complete responses (49) and CAR-NK cell-immune checkpoint inhibitor combinations significantly enhancing efficacy (50); additionally, novel microenvironmental targets such as the BMP7-SMAD1 signaling axis and spatial patterning of senescent CAFs correlate strongly with invasiveness (51, 52), collectively providing new directions for developing microenvironment-directed combination therapies.

### 2.7 Exploration of drug resistance mechanisms

Abnormal epigenetic regulation represents a key driver of drug resistance in neuroblastoma, as research identifies that the histone variant H2AFY maintains low MHC class I expression on tumor cells, conferring resistance to PD-1 immune checkpoint inhibitors; targeting H2AFY functionality restores immune recognition and enhances antitumor immunity (53). Circulating tumor cell (CTC) analysis further unveils resistance signatures in MYCN-amplified patients: approximately 93% carry high-copy MYCN and are stratified into two subtypes based on gene expression profiles—a metastasis-prone CCND1-high subtype and a chemoresistant ABCB1-high subtype (54). Additionally, therapy-resistant residual cells persist post-chemotherapy through multiple mechanisms, including MYC signaling suppression, NF-κB pathway activation, and interactions with tumor-associated macrophages (55). To address these resistance barriers, studies propose promising interventions: HDAC inhibitors overcome immune evasion by upregulating MHC-I expression to enhance CAR-T efficacy (40); YAP inhibition restores GD2 expression levels to counter immunotherapy resistance (56); and for ALK-low tumors, ALK inhibitors combined with CAR-T therapy remain effective (35). Collectively, these findings provide multifaceted and systematic solutions to tackle drug resistance in neuroblastoma treatment.

## 3 Radiomics

**Figure 1:**
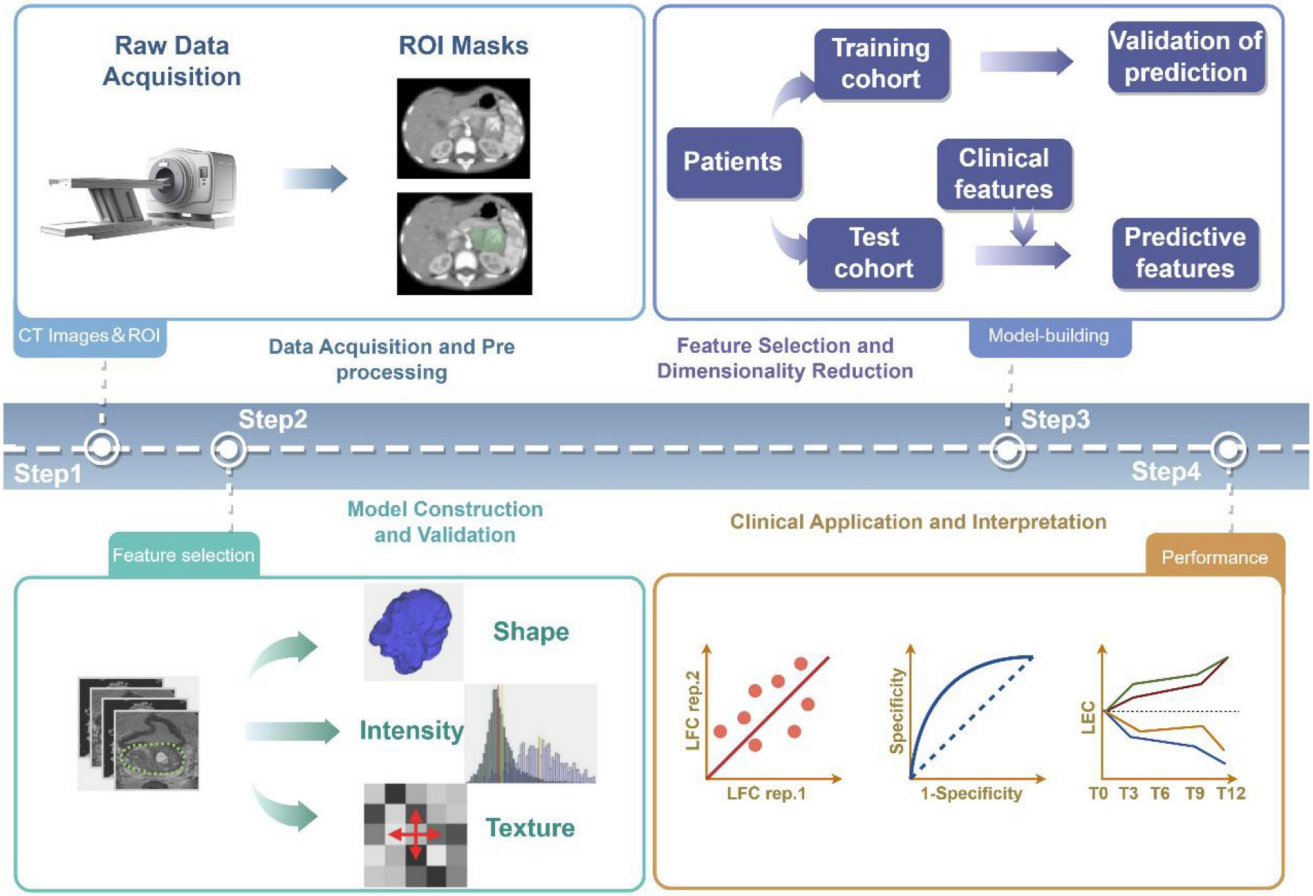
Radiomics workflow(by figdraw).

Radiomics is a technology that transforms conventional medical images into high-dimensional, quantifiable datasets. By deeply analyzing texture, morphological, and metabolic information within images, it provides noninvasive, real-time decision support for molecular subtyping, prognostic assessment, and dynamic monitoring of treatment response in neuroblastoma, with specific workflows illustrated in Figure 1. In recent years, advances in machine learning and deep learning algorithms have progressively overcome the limitations of traditional qualitative image analysis, enabling the construction of more precise predictive and classification models with broad clinical applicability. Key research progress and representative achievements are summarized in Table 2.

**Table 2:** Summary of radiomics research advances.

Author & Year	Research Objective	Imaging Modality	Sample Size	Radiomic Feature Types	Analysis Methods	Key Findings
Morin et al. (57)	Evaluate gadolinium contrast media's impact on neuroblastoma staging	MRI (T1WI-enhanced)	50	Tumor size, IDRFs (Image-Defined Risk Factors), metastasis	Kappa/Fleiss kappa statistics	Contrast media did not improve IDRF or metastasis consistency; IDRF count showed no prognostic significance
Zhao et al. (58)	Predict pathological subtypes of neuroblastoma using CT-based radiomics	CT (enhanced)	104	Morphological features (volume, shape), texture features (Gray-Level Co-occurrence Matrix)	LASSO regression, logistic regression	Combined clinical and radiomics model achieved 80.8% accuracy in distinguishing GNB/GN from NB
Wang et al. (59)	Differentiate neuroblastoma from ganglioneuroma using T2WI radiomics	MRI (T2WI)	102	Texture features (Gray-Level Size Zone Matrix)	Linear Discriminant Analysis (LDA), ROC	Age-integrated model achieved AUC = 0.963 (training) and AUC = 0.871 (testing)
Qian et al. (8)	Predict MYCN copy number status in neuroblastoma	PET/CT (18F-FDG)	104	Metabolic parameters (SUVmax, MTV), radiomic texture features	Multi-omics model (clinical + imaging), ROC	Multi-omics model distinguished MYCN wild-type vs. amplified tumors (AUC = 0.83) and MYCN gain vs. amplification (AUC = 0.95)
Qian et al. (60)	Predict MYCN amplification and 1p/11q chromosomal abnormalities	PET/CT (18F-FDG)	122	Metabolic parameters (SUVmax), radiomic texture features	LASSO regression, XGBoost algorithm	Combined clinical-radiomics model predicted MYCN amplification with AUC = 0.96 (training) and AUC = 0.92 (testing)
Tan et al. (61)	Predict MYCN amplification status using CT radiomics	CT (enhanced)	54	Morphological features (tumor margin), texture features (gray-level inhomogeneity)	XGBoost algorithm, ALE effect analysis	Model predicted MYCN amplification with AUC = 0.93 (training) and AUC = 0.88 (testing)
Ghosh et al. (62)	Predict MYCN amplification via ADC histogram analysis	MRI (DWI-ADC)	62	ADC histogram parameters (entropy, energy, uniformity)	Mann–Whitney U test, ROC analysis	MYCN-amplified tumors showed higher ADC entropy (AUC = 0.85)
Yang et al. (63)	Assess associations between IDRFs, clinicopathological features, and prognosis	CT/MRI (multimodal)	72	IDRF count and type (vascular invasion, organ infiltration)	Survival analysis (Cox model)	IDRFs ≥4 or vascular infiltration correlated with poor prognosis, but metastasis remained the dominant prognostic factor
Mansfield et al. (64)	Evaluate neoadjuvant chemotherapy's impact on IDRFs and surgical resection rates	CT/MRI (multimodal)	88	IDRF count, tumor volume change rate	Paired t-test, logistic regression	Post-chemotherapy IDRFs decreased by 2.9 per case; residual IDRFs ≤3 increased gross total resection (GTR) rate (OR=9.33)
Wang et al. (65)	Correlate post-chemotherapy IDRF changes with pathological features	CT (enhanced)	43	IDRF type (renal hilar/adjacent structure infiltration), residual IDRFs	Wilcoxon test, Spearman correlation	Post-chemotherapy IDRFs decreased (6 → 4 per case), but renal hilar infiltration showed limited improvement; residual IDRFs linked to intraoperative bleeding
Yadgarov et al. (66)	Prognostic value of metabolic heterogeneity (asphericity) in neuroblastoma	SPECT/CT (123I-mIBG)	28	Metabolic heterogeneity (asphericity), metabolic tumor volume (MTV)	Cox regression, propensity score matching	Asphericity ≥65% and MTV ≥50 mL were independent poor prognostic factors (HR = 5.32 and HR = 4.31, respectively)
Liu et al. (67)	Assess whole-tumor metabolic heterogeneity for neuroblastoma prognosis	PET/CT (18F-FDG)	95	Metabolic heterogeneity (AUC-CSH index), metabolic parameters (WMTV, WTLG)	Cox regression, ROC analysis	Whole-tumor metabolic heterogeneity (WMH) independently predicted PFS and OS (HR = 3.83), outperforming traditional metabolic parameters

### 3.1 Diagnosis and tumor characterization

Radiomics has demonstrated significant value in the precise diagnosis and tumor heterogeneity assessment of neuroblastoma. By analyzing texture, shape, and edge characteristics in MRI, CT, and PET-CT images, this technology enables quantitative characterization of spatial heterogeneity within tumors. Study (57) noted that gadolinium-based contrast media (GBCM) did not significantly improve interobserver agreement when evaluating image-defined risk factors (IDRFs), suggesting contrast-free MRI could serve as a viable alternative to ensure diagnostic quality while reducing potential side effects. Moreover, both qualitative features (e.g., tumor encasement of major vessels or adjacent organ invasion) and quantitative features (e.g., ≥4 IDRFs) within IDRFs have been confirmed as critical imaging biomarkers of tumor aggressiveness. Combined analysis of these features enhances diagnostic accuracy and clinical utility. Further research by Zhao et al. (58) revealed that a CT-based radiomics classification model achieved an overall accuracy of 80.8% in predicting three pathological subtypes of neuroblastoma. Another study indicated that comprehensive radiomics analysis of T2-weighted imaging throughout the entire tumor volume holds promise for effectively differentiating neuroblastoma (NB) from ganglioneuroblastoma/ganglioneuroma (59). Overall, radiomics is evolving from traditional morphological description to molecular-functional visualization, ultimately providing robust data-driven evidence for personalized diagnosis and therapeutic decision-making in neuroblastoma.

### 3.2 Integration with molecular subtyping data

MYCN amplification serves as a critical molecular biomarker for stratifying neuroblastoma patient subgroups, where Qian et al. (8) developed a multi-omics integration model that fuses metabolic heterogeneity parameters from 18F-FDG PET/CT imaging with clinical indices, achieving strong predictive performance in distinguishing MYCN wild-type from amplified tumors (AUC = 0.83) and excelling in discriminating MYCN gain vs. amplification status (AUC = 0.95), significantly outperforming single-modality models; Feng et al. (60) further demonstrated that pretreatment 18F-FDG PET/CT radiomic features can identify MYCN amplification while concurrently detecting key genomic aberrations like 1p deletion and 11q abnormalities, indicating metabolomic imaging can partially reflect tumor molecular biology. Although triple-phase CT radiomics showed superior MYCN prediction accuracy (61), single-phase venous CT remains clinically preferred due to its accessibility and practicality; while MRI is the primary staging modality, its radiomic models exhibit lower discrimination than CT for MYCN amplification prediction (62), suggesting multiparametric MRI techniques could enhance future molecular subtyping applications.

### 3.3 Bone marrow involvement assessment

Prediction of bone marrow metastasis via CT radiomics has achieved notable progress, as Chen et al. (6) successfully developed a risk stratification model using a multi-layer perceptron algorithm applied to 2,632 imaging features extracted from venous- and arterial-phase CT scans, achieving AUCs of 0.97 in the training cohort and 0.90 in validation; with clinical parameter integration further boosting model stability to a validation AUC of 0.91—results confirming that key radiomic signatures (including tumor heterogeneity and volume) strongly correlate with metastasis risk. This technology offers a highly efficient, noninvasive surveillance method for high-risk neuroblastoma patients, potentially reducing trauma and complications associated with conventional bone marrow biopsies while demonstrating broad clinical application prospects.

### 3.4 Risk stratification

Comprehensive assessment of IDRFs significantly enhances neuroblastoma risk stratification accuracy, as Yang et al. (63) identified that ≥4 IDRFs, vascular-related IDRFs (e.g., celiac trunk encasement), and infiltrative IDRFs (e.g., diaphragmatic invasion) serve as independent poor prognostic indicators, with predictive power particularly amplified in metastatic cases; subsequent research (64) demonstrated that neoadjuvant chemotherapy substantially reduced IDRF counts from a mean of 5.0 to 2.3 (*p* < 0.01), and when residual IDRFs post-surgery were ≤3, complete resection rates increased by 9.33-fold—highlighting the critical role of dynamic IDRF monitoring in surgical decision-making; additionally, tumors exhibiting low-to-moderate proliferative activity showed inferior chemotherapy response, underscoring the necessity of integrating molecular biomarkers like MYCN amplification status for optimized personalized risk stratification and treatment strategies.

### 3.5 Treatment response prediction

Radiomics enables dynamic chemotherapy response monitoring and surgical risk assessment, as demonstrated by Wang et al. (65) through longitudinal CT analysis revealing that neoadjuvant chemotherapy significantly reduced median IDRFs from 6 to 4 (*p* < 0.001), though certain IDRFs like renal hilum invasion showed limited improvement (*p* > 0.05); residual IDRF count exhibited moderate positive correlation with intraoperative blood loss (*r* = 0.399, *p* = 0.008) but no significant association with postoperative complications. Furthermore, study (64) confirmed that the magnitude of IDRF reduction strongly predicted gross total resection (GTR) probability—achieving >90% GTR likelihood when IDRFs decreased to ≤3 (OR = 9.33)—demonstrating that imaging-based evaluation of IDRF dynamics provides robust evidence for personalized neoadjuvant therapy optimization.

### 3.6 Prognostic prediction

Given the high heterogeneity in clinical outcomes among neuroblastoma patients—particularly high-risk cases with <50% 5-year survival—identifying reliable prognostic biomarkers is crucial for treatment optimization. Liu et al. (9) integrated CT radiomic features with artificial neural networks to develop an efficient predictive model demonstrating superior discrimination (ROC-AUC = 0.83) over conventional regression methods for evaluating mortality risk, metastatic status, and MYCN amplification; Yadgarov et al. (66) further identified tumor non-sphericity index (≥65%) and metabolic tumor volume (≥50 mL) on ^123^I-mIBG SPECT/CT as independent predictors of event-free survival (HR = 5.32 and 4.31, respectively), outperforming traditional genetic markers like MYCN amplification; additionally, Liu et al. (67) confirmed that whole-tumor metabolic heterogeneity parameters from ^18^F-FDG PET/CT independently predicted progression-free and overall survival (*p* < 0.01), with prognostic value clearly superior to conventional imaging metrics.

## 4 Pathomics

**Figure 2:**
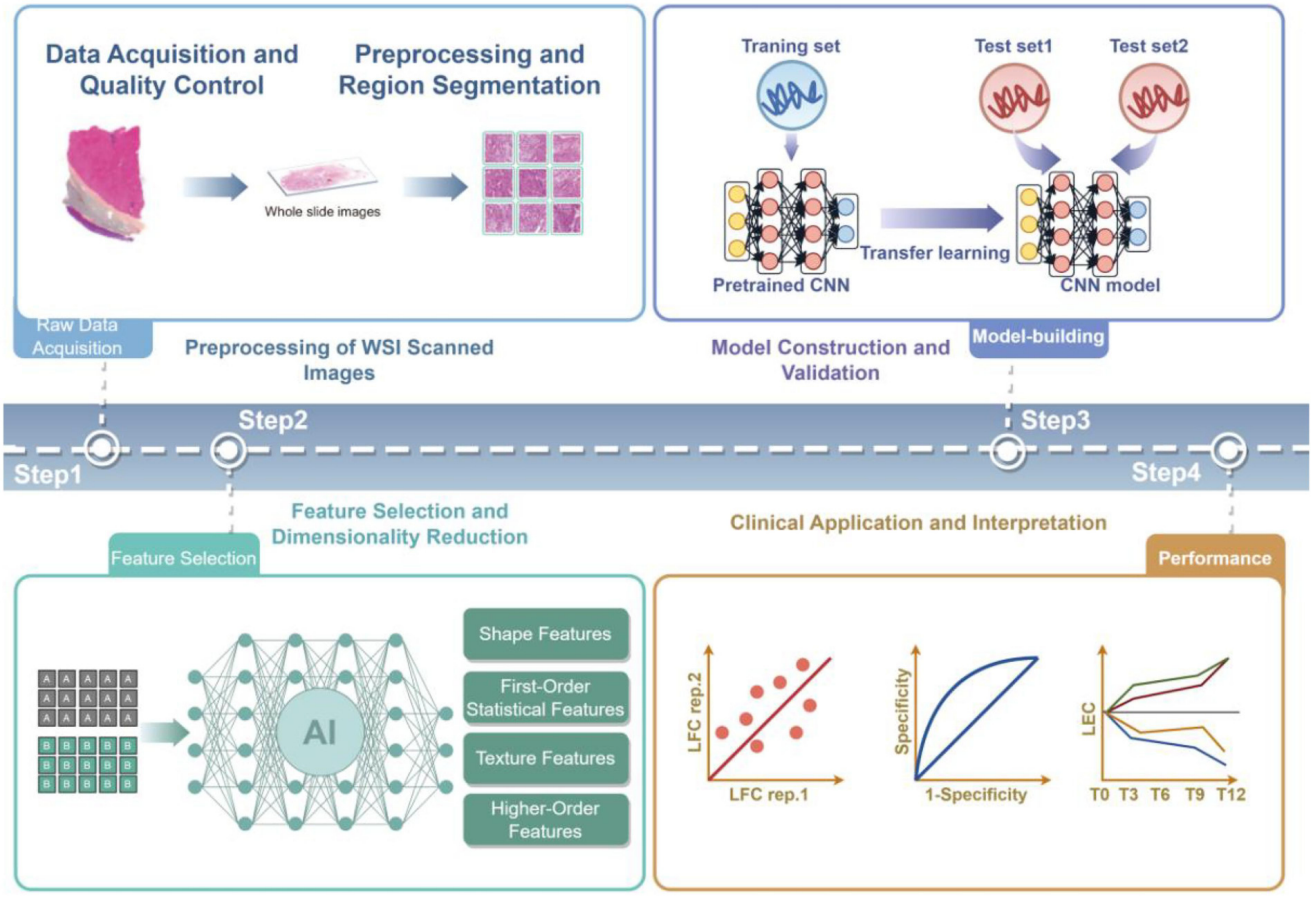
Pathomics workflow(by figdraw).

Pathomics represents an emerging technology leveraging digital pathology images to decode tumor microstructure and biological characteristics, whose standardized workflow (Figure 2) has demonstrated potential across various cancers (68) through key sequential steps: high-resolution whole-slide digitization of H&E/IHC-stained sections with chroma normalization algorithms to correct staining variations; precise segmentation of tumor regions, stroma, and necrotic zones via deep learning models like U-Net; multi-dimensional feature extraction encompassing morphological metrics (e.g., nuclear area-perimeter ratio), textural patterns (e.g., GLCM energy values), and topological properties (e.g., nuclear spatial density); subsequent feature selection using LASSO regression or graph convolutional networks to build predictive models—including SVM or deep survival networks—for molecular subtyping or prognostication; followed by SHAP-value visualization to enhance interpretability and multicenter cohort validation for assessing generalizability and clinical utility; despite significant progress in other oncology domains, pathomics research in neuroblastoma remains limited, with few published studies currently representing early-phase investigations (Table 3).

**Table 3:** Summary of advances in pathomics research.

Author(s) & Year	Research Objective	Pathology Image Type	Sample Size	Pathological Feature Types	Analytical Methods	Key Findings
Zeng et al. (77)	Evaluate prognostic impact of CD3/CD8-based Immunoscore in neuroblastoma	Immunohistochemistry (CD3, CD8, CD45RO)	244	CD3+/CD8+ T-cell density, CD45RO + memory T-cell density	Digital pathology analysis, Cox survival analysis	Low CD3-CD8- Immunoscore independently correlated with poor OS (HR = 6.39) and EFS (HR = 4.65)
Bussola et al. (79)	Quantify immune infiltration (CD3+ T cells) in neuroblastoma	CD3 IHC whole-slide images (WSI)	54	Lymphocyte density, spatial distribution	Deep learning (EUNet), topological data analysis (TDA), UMAP/HDBSCAN clustering	Model achieved mean absolute error (MAE) of 3.1 for lymphocyte density; UMAP revealed pathology-aligned spatial patterns
Stoks et al. (106)	Develop digital pathology workflow to assess ECM-targeted therapy efficacy	Immunofluorescence (Vitronectin, *α*v*β*3)	16	Vitronectin-secreting areas, αvβ3 expression intensity	Automated image segmentation, quantitative analysis	Combination therapy (Cilengitide + chemotherapy) reduced αvβ3 expression (34% vs. 66%) and suppressed tumor growth
Touioui et al. (73)	Assess prognostic value of PRAME and MCM6 protein expression	Immunohistochemistry (PRAME, MCM6)	84	PRAME and MCM6 expression intensity	Digital image quantification, Cox regression	High PRAME expression correlated with poor EFS (*p* = 0.05), indicating worse prognosis
Zormpas-Petridis et al. (71)	Develop deep learning framework for tumor heterogeneity classification on low-resolution WSI	H&E-stained and multiplex IHC WSI	73	Tumor, stroma, necrosis,lymphocyte regions	Superpixel segmentation (SLIC), convolutional neural network (CNN)	Model classification accuracy >93%, distinguishing neuroblastoma mouse genotypes (*p* < 0.05)
Ramesh et al. (72)	AI-assisted neuroblastoma classification and MYCN amplification prediction	H&E-stained WSI	197	Mitosis-karyorrhexis index, tumor differentiation	Self-supervised learning (SSL), attention-based multiple instance learning (aMIL)	Model achieved AUC > 0.85 for diagnostic subtypes, MKI, and MYCN amplification, with stable validation performance
Yu et al. (70)	Automate MKI calculation to assist pathological evaluation	H&E-stained WSI	16	Mitotic and karyorrhectic cell counts	QuPath automated counting vs. manual counting	Automated counting showed high concordance with manual results (F1-score >0.98), altering prognosis classification in 6.3% of cases
Bhardwaj et al. (69)	Validate MKI accuracy in fine-needle aspiration (FNA) samples	Cytology smears, cell block H&E staining	50	Mitotic/karyorrhectic cell density	Digital Image Visual Analysis System (DIVAS) vs. manual assessment	DIVAS achieved 96% concordance with histological MKI; INPC classification correlated with clinical outcomes (*p* = 0.029)

### 4.1 Pathomics-Assisted diagnosis

Pathomics significantly enhances precise neuroblastoma diagnosis by digitally quantifying nuclear morphology and tissue heterogeneity, exemplified by Bhardwaj et al. (69) developing the DIVAS system for automated assessment of fine-needle biopsy samples—quantifying the mitotic-karyorrhexis index (MKI) standardized pathological subtyping with 96% concordance against gold-standard histology, achieving superior inter-observer agreement (*κ* = 0.85, 98% for high-MKI samples) vs. manual evaluation; crucially, DIVAS validated MKI subtyping's prognostic significance in biopsy samples (*p* = 0.029) for the first time. Yu et al. (70) expanded digital pathology applications using the open-source platform QuPath for automated whole-slide tumor cell counting, demonstrating near-perfect human-machine concordance (F1-score > 0.98), refining MKI grading in 18.8% cases and altering risk stratification decisions for one patient. Additionally, Zormpas-Petridis et al. (71) introduced SuperHistopath, a deep learning framework integrating superpixel segmentation with CNNs to enable >93% accurate multi-class tissue recognition (tumor/stroma/necrosis) in low-resolution images within ∼5 min per slide, establishing foundations for scalable clinical implementation. These technologies thereby not only elevate diagnostic precision but also deliver practical solutions for standardized, automated analysis of minimally invasive specimens.

### 4.2 Noninvasive prediction of gene mutations

Deep learning-based multitask prediction models are effectively enhancing the efficiency and accuracy of molecular subtyping in neuroblastoma, as demonstrated by Siddhi Ramesh et al. (72) developing an attention-based multiple-instance learning (aMIL) model that leverages H&E-stained digital pathology images to noninvasively predict MYCN amplification status with exceptional discriminatory performance in external validation cohorts—the model discerns spatially heterogeneous features in tumor regions, including morphological indicators like nuclear hyperchromasia and chromatin clumping, revealing structural-genomic correlations. Concurrently, Touioui et al. (73) quantified PRAME and MCM6 protein expression through digital image analysis, identifying high PRAME expression as significantly correlated with both bone marrow metastasis and elevated mitotic-karyorrhexis index (MKI), providing novel pathways for noninvasive prediction of aggressive disease; furthermore, cross-cancer technology transfer brings breakthroughs exemplified by Bilal et al. (74), whose weakly supervised deep learning model (AUC 0.79–0.86) for colorectal cancer employs a multitask framework with strong generalizability, holding promise for predicting critical molecular events like ALK mutations or TERT rearrangements in neuroblastoma—collectively signifying the maturation of molecular prediction techniques that may ultimately enable precision subtyping without molecular assays.

### 4.3 Treatment response prediction

Pathomics demonstrates significant potential for dynamically assessing tumor sensitivity to adjuvant therapies, as Sun et al. (75) revealed that integrating pathomic and radiomic features through multimodal fusion substantially enhances radiotherapy response prediction—for instance, nuclear mitotic density and textural heterogeneity features (e.g., GLCM contrast) in H&E-stained images effectively reflect proliferative activity, while radiomic markers like ADC value heterogeneity in MRI quantify tumor hypoxia; combining these features into a unified model elevated radiosensitivity prediction AUC by over 15% compared to unimodal approaches, highlighting the superior predictive power of multi-omics integration for therapeutic response assessment.

### 4.4 Survival prognostic stratification

Traditional neuroblastoma pathological grading systems primarily rely on subjective assessment of tumor differentiation and proliferative activity by pathologists, leading to substantial interobserver variability; to address this limitation, Sertel et al. (76) pioneered a multiresolution texture analysis framework for H&E whole-slide images that quantifies Schwannian stromal development by extracting tumor-stroma spatial distribution features through gray-level co-occurrence matrices and local binary patterns—their modified k-nearest neighbor classifier achieved 88.4% accuracy in stratifying tumors into stroma-rich/stroma-poor subtypes, establishing stromal development as an independent prognostic marker and informing subsequent pathomic model designs; Zeng et al. (77) further advanced prognostic assessment by developing an Immunoscore system based on CD3+/CD8+ T-cell density, revealing CD3-/CD8- low-scoring patients faced 6.39-fold greater mortality risk independent of MYCN amplification, highlighting the microenvironment's critical role; Zormpas-Petridis et al. (71) successfully employed deep learning to classify molecular subtypes in murine models using pathological images, reinforcing genotype-phenotype correlations; Saltz et al. (78) universally validated across cancers that spatial patterns of tumor-infiltrating lymphocytes (TILs) in H&E images significantly correlated with overall survival (*p* < 0.001), offering a translational framework for neuroblastoma prognosis stratification.

### 4.5 Tumor microenvironment

Pathomics leverages spatial heterogeneity analysis to generate near single-cell resolution dynamic maps of the tumor microenvironment, enabling deeper insights into tumor-immune interplay—epitomized by Bussola et al.'s EUNet model (79), which integrates topological data analysis (TDA) with UMAP dimensionality reduction to achieve precise lymphocyte density quantification (MAE = 3.1) and identify immune subpopulations aligned with clinicopathological features; Heindl et al. (80) systematically demonstrated how spatial point pattern analysis in digital pathology deciphers TME heterogeneity, using single-cell localization to reveal topological relationships (e.g., K-function analysis of CD8+ T-cell/cancer cell clustering), advancing understanding of immune exclusion/infiltration mechanisms; Li et al. (81) utilized AI-assisted spatial correlation in pancreatic cancer to reveal that neoadjuvant therapy strengthened spatial coupling between CD8+ T-cells and tumor cells (32% increase in GLM coefficient), correlating with 45% longer survival—providing a translatable framework for neuroblastoma treatment response assessment; Wang et al.'s dual-layer graph model (82) overcame single-scale limitations by integrating cellular-level (inter-cellular distance) and tissue-level (stromal density) features to stratify breast cancer TME into three prognostic subtypes (HR = 2.1, *p* < 0.001), with spatial metrics complementing traditional grading systems; this approach is transferable to neuroblastoma for mapping spatial crosstalk between CAFs and M2-polarized macrophages in MYCN-amplified tumors, thereby pinpointing CXCL12-CXCR4 axis activation hotspots to guide spatially targeted interventions—collectively propelling TME research from static characterization toward dynamic mechanistic dissection.

## 5 Current research status of multi-omics integration

**Figure 3:**
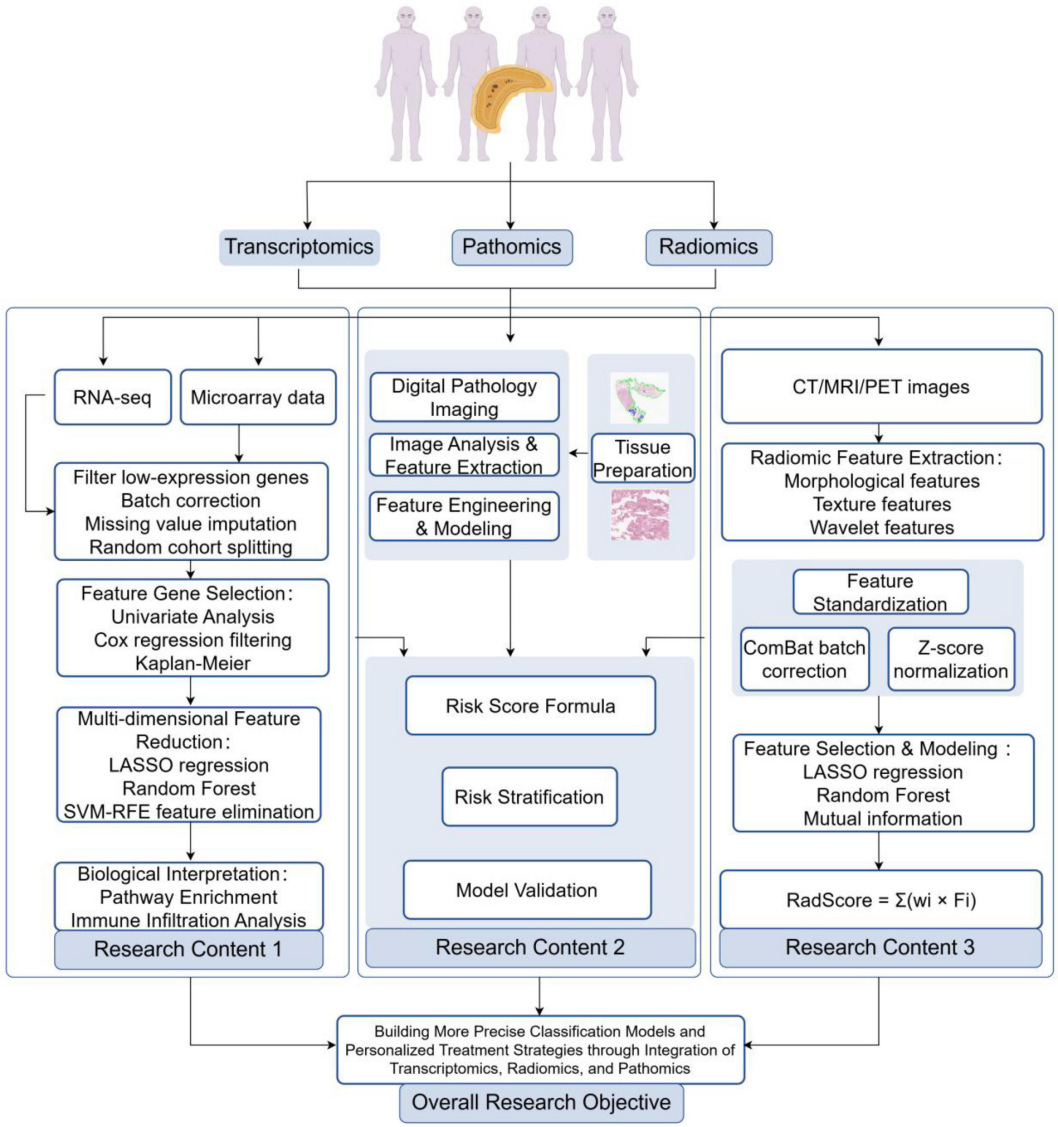
Transcriptomics, radiomics, and pathomics integration workflow(by figdraw).

Multi-omics integration serves as a core strategy for unraveling complex biological processes, providing a comprehensive analytical framework to decipher neuroblastoma's molecular heterogeneity, dynamic tumor microenvironment alterations, and therapy resistance mechanisms through systematic fusion of genomic, imaging, pathological, and clinical data (Figure 3); however, despite significant research-level advances, its translation into clinical practice faces systemic challenges across technological integration, model validation, and practical implementation (83), with key findings summarized in Table 4.

**Table 4:** Summary of advances in multi-omics research.

Author (s) & Year	Research Objective	Omics Types	Sample Size	Analytical Methods	Key Findings
Ma et al. (90)	Elucidate molecular mechanisms of rare pulmonary tumors	Transcriptomics, Radiomics, Pathomics	1	NPARS framework (integrating NGS data), multi-omics feature analysis	Identified AKT1 driver mutation and TP53 pathway inactivation, proposing post-recurrence targeted therapy strategies
Zhao et al. (88)	Predict distant metastasis in rectal cancer	Radiomics, Pathomics, Immunoscore	201	LASSO-Cox regression, nomogram construction	Combined model (C-index = 0.902) outperformed single-omics approaches; low immunoscore correlated with high metastasis risk
Feng et al. (87)	Predict overall survival in hepatocellular carcinoma	Radiomics, Pathomics	126	LASSO Cox regression, nomogram development	Radiopathomic model achieved C-index = 0.875, surpassing single-modality models
Wang et al. (86)	Predict neoadjuvant chemotherapy efficacy in nasopharyngeal carcinoma	Radiomics, Pathomics	389	Particle Swarm Optimization-Support Vector Machine (PSO-SVM)	Combined model AUC = 0.917 (internal validation), outperforming single-omics models
Wang et al. (85)	Predict post-lung metastasis outcomes in colorectal cancer	Radiomics, Pathomics	103	Convolutional Neural Network (CNN), SVM, nomogram integration	Combined model predicted OS (AUC = 0.860) and DFS (AUC = 0.875), surpassing single-omics models
Hussein et al. (28)	Identify neuroblastoma biomarkers	Pathomics, Transcriptomics	99	Similarity Network Fusion (SNF), interaction network analysis	Identified 3 transcription factors (e.g., MYCN) and 7 miRNAs as biomarkers, regulating tumor differentiation and drug resistance

### 5.1 Integration of transcriptomics and pathomics

Tumors exhibit significant spatial heterogeneity, leading to marked variations in gene expression across different regions, whereas whole-tissue pathology images provide comprehensive structural and morphological context that mitigates sampling bias inherent in genomic analysis—yet histopathological features alone often inadequately capture clinical behavior or explain biological mechanisms underlying phenotypic changes. Therefore, contextualizing multi-omics data within histological frameworks is essential for holistically understanding tumor biology and progression. For instance, Hussein et al. (28) integrated single-cell transcriptomics with pathomic features (e.g., mitotic density, chromatin heterogeneity) to reconstruct a MYCN-driven regulatory network, revealing how transcription factor TFAP2B and the miR-17-92 cluster co-regulate purine metabolism, thereby identifying three therapeutic targets that rationalize combined TFAP2B/DHODH inhibition strategies. Additionally, Liu et al. (84) combined mitotic figure density (>5/HPF) from pathomics with cell-cycle pathway scores from transcriptomics into a logistic regression model achieving 86.5% prognostic stratification accuracy, significantly outperforming the International Neuroblastoma Pathology Classification (INPC) system; cross-cancer validation (85) further confirmed that integrating weakly supervised CNN-derived histology features with gene expression profiles boosted immunotherapy response prediction (AUC increase >15%), offering critical insights for precise application of anti-PD-1/CTLA-4 inhibitors in neuroblastoma.

### 5.2 Integration of pathomics and radiomics

Radiomics harnesses advanced image processing and computer vision techniques to perform high-throughput quantification of tissue heterogeneity in medical imaging, capturing tumor characteristics at the macroscopic scale, while pathomics provides complementary quantitative insights into cellular architecture and tissue morphology at the microscopic level—their integration enables multi-scale characterization of spatial heterogeneity, forming synergistic models with enhanced representational and predictive power. Qian et al. (8) developed a PET/CT-pathomics fusion model revealing a strong correlation (*r* = 0.72) between metabolic heterogeneity (SUVmax coefficient of variation >0.35) and nuclear pleomorphism (nucleocytoplasmic ratio >0.8) in H&E sections, jointly achieving an AUC of 0.95 for MYCN amplification detection. For therapy response monitoring, Wang et al. (86) integrated MRI radiomics with pathomics in nasopharyngeal carcinoma using a particle swarm-optimized SVM model, attaining high accuracy (AUC = 0.917) for neoadjuvant chemotherapy efficacy prediction. In liver cancer research (87), arterial-phase MRI texture features combined with mitotic counts generated a combined nomogram with robust prognostic stratification (5-year OS C-index = 0.875). Wang et al.'s longitudinal analysis (65) demonstrated moderate positive correlation (*r* = 0.65) between IDRF reduction on CT and decreased M2-macrophage proportions in pathology, confirming imaging biomarkers can dynamically track immune microenvironment evolution. This multi-scale fusion methodology substantially advances comprehensive tumor profiling and provides novel frameworks for precision diagnosis, treatment assessment, and mechanistic exploration.

### 5.3 Integration of pathomics, radiomics, and transcriptomics

The multimodal integration of radiomics, pathomics, and transcriptomics enables comprehensive tumor characterization across biological scales, though such multidimensional data acquisition remains challenging with limited current research; in rectal cancer, a study (88) developed an integrated nomogram combining baseline MRI radiomics (e.g., ADC heterogeneity), pathomics (local nuclear distribution patterns), and biopsy-derived immunoscores (CD3+/CD8+ density) that successfully predicted distant metastasis risk after neoadjuvant chemoradiation (C-index = 0.902), while colorectal research (85) created a combined radiomics-pathomics nomogram (featuring tumor spiculation on imaging and stromal collagen fiber orientation entropy) demonstrating strong prognostic performance for post-lung-metastasectomy survival (OS AUC = 0.86), establishing a cross-cancer framework for monitoring neuroblastoma recurrence risk. Additionally, Braman et al. (89) proposed an innovative strategy embedding radiomic, pathomic, and genomic features to extract optimally complementary information for glioblastoma survival prediction, whereas in rare tumors like pulmonary sclerosing pneumocytoma, Ma et al. (90) integrated genomic drivers (AKT1 mutations), CT radiomics (ground-glass opacity texture), and pathomics (nucleocytoplasmic ratios) to propose novel targeted approaches—collectively providing valuable insights for investigating rare neuroblastoma subtypes and optimizing therapeutic strategies.

## 6 Challenges and future directions

### 6.1 Data heterogeneity and standardization

A core challenge in multi-omics research is data heterogeneity—variations in technical platforms, imaging parameters, and pathological staining standards introduce significant quality and feature expression biases across datasets, compromising result accuracy and reproducibility (91). For instance, registration errors (>20 μm) between spatial transcriptomics and digital pathology images can introduce >25% bias in TME analysis; multi-center studies show batch effects causing 30%–40% performance degradation during cross-institutional validation (92). To address this, standardization initiatives are underway: transcriptomics recommends uniform sequencing depth (e.g., >50,000 reads/cell for scRNA-seq), imaging protocols standardize CT slice thickness (≤1 mm), and histopathology controls H&E staining variations (e.g., pH stability within ±0.2 units). Computationally, adversarial domain adaptation tools like scGAN reduce technical bias by ∼60% in small-scale tests through cross-platform data alignment, while open-science platforms including the European Genome-phenome Archive (EGA) and NCI's Cancer Research Data Commons (CRDC) support metadata annotation and API-driven cross-institutional data integration through standardized infrastructure (93, 94).

### 6.2 Algorithm selection and model optimization

Algorithm development in multi-omics research faces two core challenges: vulnerability to overfitting in high-dimensional, small-sample settings and inadequate interpretability, limiting clinical trustworthiness. To address these, researchers have proposed innovative computational strategies. Viaud et al. (95) introduced a unified framework integrating Multiple Factor Analysis (MFA) with deep disentangled autoencoders, incorporating a Weighted Integrated Clustering Index (WICI) to balance contributions from diverse omics data—this approach validated biologically consistent stratification in neuroblastoma datasets while demonstrating robust generalization capabilities and biological relevance. Wang et al. (96) employed a network-based fusion method (PSN-SNF) for multi-omics integration, showing enhanced robustness and stability over conventional feature-level fusion in prognostic prediction tasks, substantially improving model adaptability to complex data. Feng et al. (97) developed a heterogeneous ensemble method that optimized feature subset combinations by fusing base models (decision trees, XGBoost, etc.), achieving 91.64% accuracy in survival prediction; critically, the study enhanced interpretability through Partial Dependence Plots and decision rule extraction techniques, advancing the development of high-accuracy, high-interpretability clinical decision systems.

### 6.3 Clinical translation and personalized therapy

Prediction models for neuroblastoma predominantly rely on retrospective static data and lack real-time dynamic monitoring capabilities, with radiomics typically requiring 2–4 weeks per assessment—insufficient for tracking rapid tumor progression; while traditional invasive biopsies carry >15% complication rates, limiting serial monitoring utility. Liquid biopsy breakthroughs now enable therapeutic response evaluation within 72 h via ctDNA methylation panels and exosomal miRNA detection (sensitivity >85%) (98). Portable ultrasound systems (e.g., Butterfly iQ+) integrated with edge-computing AI algorithms can analyze tumor hemodynamics in real-time (e.g., resistance index >0.8 indicating high-risk lesions) at 10% of MRI costs, enhancing accessibility in primary care settings. Emerging technologies include electrochemical aptamer sensors achieving aM-level sensitivity for neuroblastoma-associated miRNAs (miR-181/184) to refine minimal residual disease monitoring (99). Noninvasive diagnostic advances include Du et al.'s plasma-based metabolic-transcriptomic classifier (S100A9/CDK2/UNC5D) enabling high-risk neuroblastoma early screening (AUC = 0.837) (100) and Wang et al.'s multi-omic revelation that mitochondrial inhibitor Mdivi-1 induces dual tumoricidal mechanisms through serine metabolic rewiring and PSMA3 subunit dephosphorylation (101). Target discovery highlights include Hamilton et al.'s identification of super-enhancer-driven DLK1 for antibody-drug conjugate ADCT-701 development (specific cytotoxicity in xenografts) (102), and Wang et al.'s multi-task learning framework integrating gene expression/DNA methylation for survival prediction (AUC > 0.85) (103). Multi-omics prognostic tools feature Sun et al.'s nomogram (clinico-pathological features + NSE biomarker, C-index = 0.824) (104) and Watanabe et al.'s methylation-metabolomic stratification identifying ultrahigh-risk 11q-del/MYCN-amplified patients responsive to PHGDH inhibitor/arginine deiminase synergy (105). Collectively, advances in multi-omics integration, AI diagnostics, and novel biomarkers are accelerating neuroblastoma management toward real-time, precise, and personalized therapeutic interventions.

### 6.4 Future directions

The next generation of multi-omics technologies is breaking through traditional detection limitations, enabling three-dimensional dynamic profiling of tumor microenvironments across spatial and temporal dimensions, while AI-driven data augmentation combined with liquid biopsy provides viable solutions for small-sample modeling and dynamic disease monitoring; in personalized therapy, future approaches must advance from static subtyping toward dynamically feedback-guided precision interventions, where digital twin technology simulates patient treatment responses and CRISPR-based functional genomic screening identifies critical resistance targets—coupled with metabolic reprogramming strategies—to achieve precise tumor targeting and individualized modulation; accelerating clinical translation requires open-source platform sharing of high-quality pretrained models and establishing unified interoperability standards for multi-omics data; simultaneously, implementing patient-centric participatory research models that integrate patient needs and data feedback into development cycles will substantially enhance clinical relevance and translational efficiency of research outcomes.

## 7 Conclusion

Recent progress in single-omics fields like transcriptomics, radiomics, and pathomics has greatly enhanced understanding of neuroblastoma. These technologies have revealed molecular subtypes, tumor microenvironment characteristics, drug resistance mechanisms, and potential therapeutic targets. However, single-omics approaches have limitations in fully grasping tumor complexity, prompting the emergence of multi-omics integration. This integrated method combines gene expression, imaging features, and pathological data, overcoming individual technology constraints. It allows for more accurate classification models and personalized treatment strategies. The approach holds great promise for improving early diagnosis, prognosis prediction, treatment response monitoring, and personalized therapy for neuroblastoma, potentially enhancing clinical outcomes for children.
